# Small Bowel Perforation as a Postoperative Complication from a Laminectomy

**DOI:** 10.1155/2015/378218

**Published:** 2015-07-08

**Authors:** Robert H. Krieger, Katherine M. Wojcicki, Andrew C. Berry, Warren L. Reuther, Kendrick D. McArthur

**Affiliations:** ^1^Kansas City University of Medicine and Biosciences (KCU), 1750 E. Independence Avenue, Kansas City, MO 64106, USA; ^2^Department of Internal Medicine, University of South Alabama, Mobile, AL 36608, USA; ^3^Department of Radiology, West Palm Hospital, West Palm Beach, FL 33407, USA; ^4^Department of Surgery, West Palm Hospital, West Palm Beach, FL 33407, USA

## Abstract

Chronic low back pain is one of the leading chief complaints affecting adults in the United States. As a result, this increases the percentage of patients that will eventually undergo surgical intervention to alleviate debilitating, chronic symptoms. A 37-year-old woman presented ten hours postoperatively after a lumbar laminectomy with an acute abdomen due to the extraordinarily rare complication of small bowel injury secondary to deep surgical penetration.

## 1. Introduction

In the United States, back pain remains the second most common reason why patients visit a clinician and up to 84 percent of adults will eventually endure low back pain [1, 2]. Approximately 250,000–500,000 people in the United States have symptoms of spinal stenosis, and although many patients remain asymptomatic to mildly symptomatic, the prevalence of symptomatic back pain is significant enough to warrant neurosurgical intervention and case volume will remain steady [3]. Cohort studies of individuals with lumbar spinal stenosis (LSS) demonstrated that 30 percent of the patients subsequently requested surgical treatment after initially choosing nonsurgical, medical management [4, 5]. As one would expect, surgery to the vertebral column is not without risk and although ventral perforation is rare, injury to the retroperitoneal vessels is the most common, serious, complication in this scenario [6]. However, this case illustrates one of the rare instances where the small bowel was injured during a lumbar laminectomy and highlights the importance of recognizing the acute abdomen as a potentially lethal complication of a laminectomy or discectomy. Furthermore, a review of the literature revealed that this uncommon complication is more likely during a discectomy when compared to a laminectomy, which makes our reported case an exceptionally rare report [7].

## 2. Case Report

A 37-year-old female, postlaminectomy status for the treatment of lumbar back pain, presented with unrelenting abdominal pain 10 hours after the procedure. The patient denied any nausea, vomiting, diarrhea, fevers, or chills at that time, but due to the acute nature of the pain it was evident to her that she had either developed some type of postoperative complication or was experiencing a nascent abdominal pathology that would require immediate medical attention. The patient's past medical history was significant for chronic back pain and lumbar disc disease, with no significant surgical history prior to the laminectomy. Vital signs upon admission and consultation were stable and the patient was afebrile. Physical exam revealed a well-nourished, well-developed female that was alert and oriented albeit in obvious discomfort. The patient's physical exam was unremarkable except that her abdomen was exquisitely tender to palpation without any distension and there was significant tympany upon percussion. The electrolyte profile was essentially normal, but total bilirubin was elevated at 2.1 mg/dL. Urine pregnancy test was negative and the complete blood count showed an elevated white blood cell count of 15.4 × 10^3^/*μ*L, with a hemoglobin and hematocrit of 12 g/dL and 34%, respectively.

**Figure 1:**
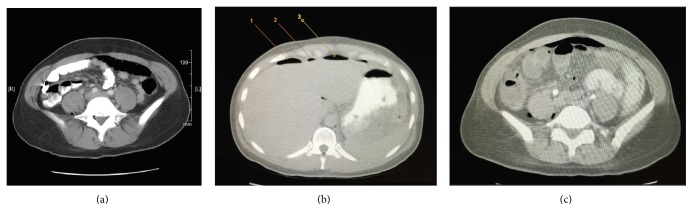
Computed tomography (CT) of the abdomen/pelvis demonstrates the progression of intra-abdominal injury and free air accumulation. (a) Transaxial view highlighting the accumulation of air in the spinal canal, psoas muscles, and the retroperitoneum, secondary to deep penetration and injury of the small bowel during the laminectomy. (b) This axial view demonstrates three distinct locations (labeled 1, 2, and 3) of intra-abdominal free air found on a follow-up CT of the abdomen/pelvis. (c) Axial view depicting extraluminal air anterior to the bowel, as well as air within the mesentery and posterior to the right psoas muscle.

Computed tomography (CT) of the abdomen and pelvis showed evidence of retroperitoneal air, which seemingly tracked back into the spinal canal (Figure 1(a)). In addition, there was free air seen intraperitoneally without an obvious source or evidence of an inflammatory process in her abdomen at that time (Figure 1(b)). It was suspected that the retroperitoneal air may be secondary to deep surgical penetration into the small bowel during the laminectomy and the patient was initially treated expectantly with intravenous antibiotics and observation. On hospital course days two and three the patient began to spike and maintain fevers, with air continuing to appear in the psoas muscles and significant air within the peritoneal cavity. The follow-up CT showed that the patient had developed ascites suggesting a small bowel perforation until proven otherwise (Figure 1(c)). The patient was taken to the operating room for emergent laparoscopic exploration.

**Figure 2:**
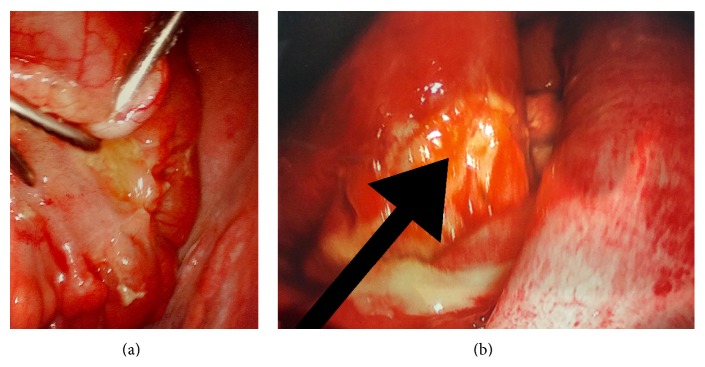
Laparoscopic views of the inflamed and perforated small bowel photographed prior to the conversion to laparotomy. (a) The photograph demonstrates a highly erythematous, inflamed segment of small bowel with fibrinous exudation and inflammation adjacent to the instrument. (b) The arrow pointing to the site of perforation located at the approximate jejunal-ileal junction.

After dissecting through the subcutaneous tissue and the anterior rectus fascia in a standard fashion, the Hassan trocar was inserted and a laparoscopic view of the abdomen was undertaken. There was a marked amount of seropurulent fluid extending from the right and left colic gutters down to the Pouch of Douglas. The appendix was visualized and appeared normal; however, while running the small bowel, beginning at the terminal ileum, a significant amount of inflammatory and fibrinous exudate was discovered (Figure 2(a)). Upon approaching the proximal ileum and jejunum, an area of perforation was identified as evidenced by where the omentum had adhered to the small bowel (Figure 2(b)). With very light manipulation, enteric contents exuded from the small bowel and the procedure was converted to an open exploratory laparotomy. After repairing the enterotomy with interrupted Vicryl sutures and reinforcing it with silk suture in a Lembert fashion, the abdomen was irrigated copiously. Other than the jejunal-ileal perforation, no other intra-abdominal pathologies were noted during exploration. The patient's postoperative hospital course was unremarkable and she tolerated the small bowel repair without complication.

## 3. Discussion

When considering the supportive anatomy of the vertebral column, specifically the annulus fibrosus and the anterior longitudinal ligament, it is not inconceivable that ventral perforation is a rather rare complication of laminectomy. In a study of 30,000 lumbar discectomies, there was a reported ventral perforation rate of 0.016% [8] and when reviewing another study of documented cases it appears that this would be even less common during a laminectomy [7]. This seems logical that laminectomies would be a rarer cause of anterior perforation as the lamina is located dorsally on the vertebral body and the bowel and retroperitoneal vasculature lies anterior to the vertebral body. The most acute, life-threatening complication from an anterior perforation would be due to an intraoperative vascular injury, which may be evident by brisk bleeding, hypotension, or shock. In fact, hemorrhage of the large retroperitoneal vessels is the most common complication due to anterior perforation of the vertebral column [9–11]. Due to the pressure in the common iliac arteries, the most commonly injured vessels during lumbar laminectomy or discectomy, an acute vascular injury is likely to be quickly recognized by the surgical team. However, in the few reported cases of small bowel injury, the patients will typically begin experiencing severe abdominal pain by the second postoperative day, which was consistent with our patient's hospital course [12]. Therefore, it is imperative to have a high index of suspicion for bowel perforation when a postoperative laminectomy or discectomy patient develops peritoneal signs.

The incidence of intestinal injury following lumbar discectomy was reported, in a large-scale study with 68,329 patients, to be 0.0015% by the German Society of Neurological Surgery [8]. Thorough literature search identified this as the 16th reported case of small bowel injury occurring after a discectomy or laminectomy, dating back to the first reported case by Harbison in 1954 [7, 13, 14]. Of all of the cases discovered in the review of the literature, we believe that this is the 2nd reported case of small bowel injury after a laminectomy [7]. The most likely mechanism by which the small bowel may be penetrated is due to the root of the mesentery arising from the anterior vertebral column at approximately L2, traveling obliquely and terminating at the right sacroiliac joint. When the patient is prone during the operation, segments of the small bowel may appear anterior to the lumbar vertebral column [6]. This mechanism, along with deep surgical penetration during laminectomy, is precisely the means by which our patient had her jejunal-ileal junction perforated. A delay in diagnosis is associated with high morbidity and mortality rate after bowel injury, especially with small bowel perforation. Exploratory laparoscopy or laparotomy followed by repair via suture or resection and anastomosis is essential [13, 15]. In one of the earlier reported cases of bowel perforation during microscopic discectomy, the patient was not diagnosed until after 48 hours and the delay resulted in peritonitis, sepsis, and death of the patient [16]. In our case, the surgical team was consulted within one postoperative day and the patient was rapidly diagnosed which prevented her from developing septic shock and a potentially fatal outcome.

Neurosurgical intervention in the vertebral column will remain common as long as chronic low back pain is a leading chief complaint in the United States. While it is not a typical complication, perforation of the viscus is associated with a high mortality rate; therefore, the surgeon must maintain a high index of suspicion when a patient presents with an acute abdomen after lumbar spinal surgery and be prepared to emergently surgically diagnose and correct the injury in the operating room.
